# Resveratrol-Enriched Rice Callus Extract Inhibits Oxidative and Cellular Melanogenic Activities in Melan-A Cells

**DOI:** 10.3390/antiox13060625

**Published:** 2024-05-21

**Authors:** Chaiwat Monmai, Jin-Suk Kim, So-Hyeon Baek

**Affiliations:** Department of Agriculture Life Science, Sunchon National University, Suncheon 59722, Republic of Korea; bbuayy@gmail.com (C.M.); kimjs6911@naver.com (J.-S.K.)

**Keywords:** resveratrol, transgenic rice, MITF, tyrosinase, hyperpigmentation

## Abstract

The excessive production of melanin can cause skin diseases and hyperpigmentation. In this study, resveratrol contained in Dongjin rice seed (DJ526) was increased through callus induction. The antioxidant capacity of resveratrol-enriched rice callus was evaluated using the ABTS radical scavenging method and was equivalent to that of vitamin C. DJ526 rice callus extract significantly increased antioxidant activities in a concentration-dependent manner. The anti-melanogenesis effects of DJ526 rice callus extract were also evaluated in melan-a cells. Resveratrol-enriched rice callus extract significantly (i) decreased the size and number of melanin-containing cells, (ii) suppressed the activity of cellular tyrosinase and melanin content, (iii) downregulated the expression of microphthalmia-associated transcription factor, tyrosinase, tyrosinase-related protein-1, and tyrosinase-related protein-2, (iv) increased the expression of phosphorylated extracellular signal-regulated kinase 1/2 and protein kinase B, and (v) inhibited the activation of phosphorylated p38 in melan-a cells. From the above observations, DJ526 rice callus extract showed strong antioxidant and anti-melanogenesis activity at the concentration test. These findings indicate the potential of resveratrol-enriched rice callus as a novel agent for controlling hyperpigmentation.

## 1. Introduction

Melanogenesis is the process of melanin production by melanocytes [1]. Melanin is a pigment and a defense mechanism of skin tissue against external stimuli such as ultraviolet radiation (UV) [2]. Several enzymes are involved in melanogenesis, including tyrosinase, tyrosinase-related protein (TRP)-1, and TRP-2, which are controlled by microphthalmia-associated transcription factor (MITF) [3,4,5]. Additionally, the expression of MITF is associated with the regulation of mitogen-activated protein kinases (MAPKs) and protein kinase B (Akt) [6,7,8]. Suppression of phosphorylated (p)-ERK 1/2 and p-Akt prevents MITF degradation. Conversely, activation of p-p38 MAPK promotes MITF activity. These suppressions and activations lead to the upregulation of tyrosinase, TRP-1, and TRP-2, resulting in increased melanin production [9,10,11]. Although melanin production protects the skin tissue from UV damage, overproduction can cause several problems such as darker patches on the skin, hyperpigmentation, postinflammatory melanoderma, freckles, melasma, and skin discolorations [12,13,14,15,16].

Resveratrol is a polyphenolic compound found in various plants such as plums, berries, grapes, and peanuts [17,18,19,20,21,22]. Many biochemical properties of resveratrol and its derivative, piceid, have been demonstrated to be potentially useful agents for human health, including preventing skin aging and pigmentation [23,24,25], antioxidant [26,27], anti-inflammatory [26,28,29,30], antitumor, antibacterial, and antifungal properties [31,32,33]. Additionally, oral administration of resveratrol reduced UV-induced skin edema and wrinkles in ICR mice through the Nrf2/HO-1 signaling pathway [34]. Notably, resveratrol and oxyresveratrol inhibit tyrosinase activity [35,36,37,38]. 

We developed resveratrol-enriched rice seeds by introducing *Arachis hypogaea* stilbene synthase from the pods of the peanut cultivar Palkwang into Dongjin rice (DJ) [39]. Subsequently, we evaluated the resveratrol content in the seeds [40] and observed that the resveratrol and piceid content in the resveratrol-enriched rice seeds are 2.605 ± 0.001 μg/g dry weight and 4.724 ± 0.02 μg/g dry weight, respectively. However, germinated seeds of resveratrol-enriched rice showed higher resveratrol and piceid content (3.230 ± 0.060 μg/g dry weight and 16.879 ± 0.024 μg/g dry weight, respectively). Therefore, we improved the resveratrol and piceid content in these rice seeds by callus induction [41]. In DJ526 rice callus extract, the piceid content was 85.43 ± 3.44 μg/g dry weight, and the resveratrol content was 3.94 ± 0.02 μg/g dry weight. Although the resveratrol and piceid content increased in DJ526 rice callus extract, their antioxidant and anti-melanogenesis activities need to be investigated. The increase in resveratrol content in DJ526 rice callus must preserve the antioxidant and anti-melanogenesis activity as well. The resveratrol content of rice seeds may vary annually depending on the growing area or growing environment. Therefore, we have developed the DJ526 rice callus for plant factories to develop a biomaterial with a stable resveratrol content and antioxidant and anti-melanogenesis properties. Therefore, this study investigates the antioxidant and anti-melanogenesis effect of DJ526 rice callus extract, which has improved accumulated content of resveratrol and piceid in melan-a cells.

## 2. Materials and Methods

### 2.1. Treatments

The DJ and DJ526 rice callus extracts were prepared as in the previous experiment [41]. Briefly, DJ and DJ526 seeds were sterilized, were induced in 2N6 medium for 3 weeks, and cultured in 2MS-NO_3_-free liquid medium for 10 days. The DJ and DJ526 extracts were isolated from dried callus using 80% methanol. The treatments were prepared at concentrations of 10, 25, 50, and 100 mg/mL in DMSO. The content of piceid and resveratrol in the rice seed extracts was quantified using high-performance liquid chromatography (HPLC) analysis. Concisely, the extract was mixed with 80% methanol, sonicated, and the supernatant was collected by centrifugation. After being filtered, the supernatant was analyzed for the content of piceid and resveratrol using a Water e2695 HPLC system (Water, Milford, MA, USA). Only DJ526 rice callus showed detectable peaks of piceid and resveratrol, with contents measured at 85.43 ± 3.44 µg/g dry weight and 3.94 ± 0.02 µg/g dry weight, respectively [41].

### 2.2. ABTS Radical Scavenging Ability

The 2,2-Azino-bis-3-ethylbenzothiazoline-6-sulfonic acid (ABTS) radical scavenging ability (ABTS^•+^) was evaluated to assess the antioxidant activity of DJ and DJ526 rice callus extracts following the procedures described previously [42]. ABTS (Roche, Basel, Switzerland) and potassium persulfate (Sigma-Aldrich, St. Louis, MO, USA) were prepared at concentrations of 14.0 mM and 4.8 mM, respectively. The ABTS and potassium persulfate solutions were mixed at a 1:1 ratio (*v*/*v*) to generate the ABTS radical cation (ABTS^•+^), which was then incubated for 16 h at room temperature in the dark. The ABTS^•+^ working solution was subsequently prepared to obtain an absorbance value of 0.70 ± 0.02 at 734 nm in 100% ethanol. DJ and DJ526 rice callus extracts at various concentrations were incubated with the ABTS^•+^ working solution at a ratio of 1:100 (*v*/*v*), with distilled water serving as the control. Various concentrations of arbutin, an antioxidant agent which reduces ROS levels by directly scavenging free radicals [43,44,45], were used as the positive control. After a 7 min incubation, the absorbance of the solution was measured at 734 nm. The ABTS radical scavenging ability (%) was calculated using the following Formula (1).

(1) $$\mathrm{ABTS}\;\mathrm{radical}\;\mathrm{scavenging}\;\mathrm{ability}\;(\%)\;=\left(\frac{A_{734\;}\mathrm{of}\;\mathrm{control}-A_{734}\;\mathrm{of}\;\mathrm{treatment}\;}{A_{734}\;\mathrm{of}\;\mathrm{control}}\right)\times \;100,$$

where A_734_ represents the value of an absorbance at 734 nm.

### 2.3. Vitamin C Equivalent Antioxidant Capacity and IC50

The vitamin C (ascorbic acid; Sigma-Aldrich, St. Louis, MO, USA) equivalent antioxidant capacity (VCEAC) of DJ and DJ526 rice callus extracts was investigated by generating a standard curve of various concentrations of ascorbic acid (*X*-axis) against ABTS radical scavenging values (*Y*-axis). The VCEAC was calculated using Formula (2) (Figure S1).

(2) $$\mathrm{VCEAC}\;(\mathrm{mg}/g\;\mathrm{dry}\;\mathrm{weight})=\frac{Y\;+\;18.926}{2.795},$$

where Y represents the ABTS radical scavenging value.

The standard curve from the graph plotting the ABTS radical scavenging value (*X*-axis) against various treatment concentrations (*Y*-axis) was used to calculate the concentration of DJ and DJ526 rice callus extracts required to inhibit the ABTS+ concentration by 50% (IC50). The X-value of the regression equation was substituted with 50 to calculate the IC50 of the extracts (Y). 

### 2.4. Melan-A Cell Viability

Melan-a cells were cultured in a cell culture medium composed of 10% fetal bovine serum (Gibco™ Thermo Fisher Scientific, Inc., Waltham, MA, USA), 1% penicillin/streptomycin (Hyclone Laboratories, Inc., Logan, UT, USA), and 20 mM 12-*O*-tetradecanoylphorbol-13-acetate (TPA; Sigma-Aldrich, St. Louis, MO, USA) in an incubator at 37 °C under 5% CO_2_. Cells were seeded at a density of 2 × 10^4^ cells per well in a 96-well plate and incubated for 24 h at 37 °C under 5% CO_2_. Subsequently, the culture medium was replaced with various concentrations of extracts (DJ and DJ526). The cell culture medium and 0.1% DMSO served as negative and model controls, respectively. Arbutin, an effective agent for treating for hyperpigmentation in the cosmetics industry [46] which has been reported to be effective as a dark spot prevention and skin-lightening agent [43,47], was used as a positive control for this experiment. After 72 h incubation, the culture medium was replaced with 110 µL of EZ-Cytox (DoGenBio, Seoul, Republic of Korea) working solution (diluted 1:10 (*v*/*v*) in 1 × PBS). The plate was incubated at 37 °C for an additional 4 h. Then, 100 µL of the solution was transferred to a new 96-well plate and the optical density was measured at 450 nm using a SpectraMax^®^ ABS Plus Microplate Reader (Molecular Devices, San Jose, CA, USA). The cell viability ratio was calculated according to Formula (3).

(3) $$\mathrm{Cell}\;\mathrm{viability}\;\mathrm{ratio}\;(\%)=\frac{\mathrm{Absorbance}\;\mathrm{at}\;450\;\mathrm{nm}\;\mathrm{of}\;\mathrm{treatment}}{\mathrm{Absorbance}\;\mathrm{at}\;450\;\mathrm{nm}\;\mathrm{of}\;\mathrm{control}}\;\times \;100,$$

### 2.5. Melanin Content

The cells were seeded at a density of 5 × 10^5^ cells/well in a 6-well plate and incubated at 37 °C under 5% CO_2_ for 24 h. Various concentrations of DJ and DJ526 rice callus extracts were then added to the cells. The plate was further incubated for 72 h at 37 °C under 5% CO_2_. Subsequently, the cells were collected using trypsin-EDTA (Welgene, Gyeongsangbuk-do, Republic of Korea) followed by centrifugation at 1200 rpm for 3 min. Cells from each treatment group were resuspended at 1 × 10^5^ cells in a 1.5 mL tube. Then, the cells were disrupted by incubation with 1 N NaOH solution at 80 °C for 4 h. This experiment was performed in triplicate. The resulting solution was then measured for absorbance at 405 nm, and melanin content was assessed using Formula (4).

(4) $$\mathrm{Melanin}\;\mathrm{content}\;(\%)=\frac{\mathrm{Absorbance}\;\mathrm{at}\;405\;\mathrm{nm}\;\mathrm{of}\;\mathrm{treatment}}{\mathrm{Absorbance}\;\mathrm{at}\;405\;\mathrm{nm}\;\mathrm{of}\;\mathrm{control}}\;\times \;100,$$

### 2.6. L-DOPA Staining

Cells were seeded at a density of 2 × 10^4^ cells/well and treated with various concentrations (10, 25, 50, and 100 µg/mL) of DJ and DJ526 rice callus extracts. The experiment was performed as previously described [42,48]. After 72 h of incubation, cells were washed with 1× PBS and fixed with 10% formaldehyde (Sigma-Aldrich, St. Louis, MO, USA) at room temperature for 20 min. Subsequently, cells were washed again with 1× PBS and stained with 2 mg/mL of L-DOPA (100 µL; Sigma-Aldrich, St. Louis, MO, USA) at 37 °C for 3 h. Then, the L-DOPA solution was discarded, and the cells were washed five times with 1× PBS. The cells were then allowed to dry at room temperature, and the pigmentation of melan-a cells was imaged using an IM-3 series microscope (Optika, Bergamo, Italy).

### 2.7. Cellular Tyrosinase Activity

The tyrosinase activity assay was performed as previously described [42]. Briefly, the treated cells were collected and disrupted with 1% Triton X-100 (R&D Systems, Inc., Minneapolis, MN, USA) in 0.1 M sodium phosphate buffer (pH 6.8). The cells were lysed on ice for 30 min, followed by centrifugation at 13,000 rpm and 4 °C for 30 min. The supernatant was transferred to a new microtube, and the protein concentration was measured using the Bradford method (WELGENE, Inc., Gyeongsangbuk-do, Republic of Korea). The protein concentration from each treatment group was adjusted to 40 µg with lysis buffer in a total volume of 80 µL. The protein was then incubated with 20 µL of L-DOPA (2 mg/mL) at 37 °C for 1 h. After incubation, the absorbance of the solution was measured at 475 nm, and the cellular tyrosinase activity was calculated using Formula (5).

(5) $$\mathrm{Cellular}\;\mathrm{tyrosinase}\;\mathrm{activity}\;(\%)=\frac{\mathrm{Absorbance}\;\mathrm{at}\;475\;\mathrm{nm}\;\mathrm{of}\;\mathrm{control}\;-\;\mathrm{Absorbance}\;\mathrm{at}\;475\;\mathrm{nm}\;\mathrm{of}\;\mathrm{treatment}}{\mathrm{Absorbance}\;\mathrm{at}\;475\;\mathrm{nm}\;\mathrm{of}\;\mathrm{control}}\;\times \;100,$$

### 2.8. Morphological Appearance

The treated cells were evaluated for morphological appearance using a Fontana-Masson kit (BIOGNOST, Ltd., Zagreb, Croatia) according to the manufacturer’s instructions. The number of melanin-containing cells was counted from a total of 1000 cells (both melanin-containing and non-melanin-containing cells) under an IM-3 series microscope. A total of 100 melanin-containing cells were randomly counted and categorized into 4 groups based on their morphological appearance using a scoring system (Table S1) described by Rodboon et al. [49].

### 2.9. Gene Expression Quantification 

The treated cells were lysed using TRI reagent™ (Invitrogen, Waltham, MA, USA). The lysate was transferred into a 1.5 mL tube and mixed with 200 µL of chloroform. After centrifugation at 13,000 rpm and 4 °C for 10 min, the upper phase of the solution was collected. Total RNA was precipitated with 100% isopropanol and harvested by centrifugation at 13,000 rpm and 4 °C for 10 min. The resulting total RNA was dissolved in nuclease-free water. RNA quantity and quality were determined using a SpectraMax^®^ ABS Plus Microplate Reader (Molecular Devices, San Jose, CA, USA). RNA quality was assessed based on the absorbance ratio at 260/280 nm and 260/230 nm of the extracted RNA, with an acceptable range typically falling between 1.80 and 2.00 (Table 1). 

The first strand of cDNA was synthesized using 500 ng of RNA and the Power cDNA Synthesis Kit (Intron Biotechnology, Seongnam-si, Republic of Korea). The qPCR reaction consisted of 5 ng of cDNA template and 0.375 µM of each primer in 20 µL RealMOD™ GreenW^2^ 2× qPCR Mix (Intron Biotechnology). The specific primer sets used in this experiment are as follows: *MITF* (NM_001113198.2) forward primer: AGC GTG TAT TTT CCC CAC AG and reverse primer: CCT TAG CTC GTT GCT GTT CC; *tyrosinase* (D00131.1) forward primer: CCA GAA GCC AAT GCA CCT AT and reverse primer: CCA GAT ACG ACT GGC CTT GT; *TRP-1* (NM_031202.3) forward primer: TCT GGC CTC CAG TTA CCA AC and reverse primer: TCA GTG AGG AGA GGC TGG TT; *TRP-2* (X63349.1) forward primer: ACC CTG TGT TTG TGG TCC TC and reverse primer: GTT GCT CTG CGG TTA GGA AG; *GAPDH* (NM_001289726.2) forward primer: AAC TTT GGC ATT GTG GAA GG and reverse primer: ACA CAT TGG GGG TAG GAA CA. The qPCR reaction was run on a CFX Connect Real-Time PCR system (Bio-Rad, Hercules, CA, USA). The qPCR conditions are shown in Table 2. Expression levels were calculated as fold changes relative to parallel control groups treated with treatment medium alone using CFX Maestro software (Bio-Rad CFX Maestro 1.1; Bio-Rad, Hercules, CA, USA).

### 2.10. Western Blot

Cells were treated with various concentrations of DJ and DJ526 at 37 °C and 5% CO_2_ for 72 h. Subsequently, cells were harvested with trypsin-EDTA at 37 °C for 5 min and then centrifuged at 13,000 rpm for 1 min. The collected cells were lysed with RIPA buffer (GeneAll Biotechnology, Seoul, Republic of Korea) containing 1% protease inhibitor (Bio-Medical Science Co., Ltd., Seoul, Republic of Korea) on ice for 30 min. After centrifugation at 13,000 rpm and 4 °C for 30 min, the supernatant was collected. Protein concentrations were determined using the Bradford method and compared with a bovine serum albumin standard curve (Figure S2). 

Thirty micrograms of protein from each treatment group were separated by SDS–polyacrylamide gel electrophoresis. The separated proteins were then transferred onto a nitrocellulose membrane. The membrane was washed with 1× TBST and blocked in blocking solution (5% skim milk in 1× TBST) at room temperature for 2 h. Subsequently, the membrane was incubated overnight at 4 °C with primary antibodies specific to MITF (1:2000; Santa Cruz Biotechnology, Dallas, TX, USA), tyrosinase (1:2000; Santa Cruz Biotechnology), TRP-1 (1:2000; Santa Cruz Biotechnology), TRP-2 (1:2000; Santa Cruz Biotechnology), ERK 1/2 (1:2000; Cell Signaling, Danvers, MA, USA), phosphorylated (p)-ERK 1/2 (1:2000; Cell Signaling), Akt (1:2000; Santa Cruz Biotechnology), p-Akt (1:2000; Cell Signaling), p38 MAPK (1:2000; Santa Cruz Biotechnology), p-p38 MAPK (1:2000; Cell Signaling), and GAPDH (1:5000; Santa Cruz Biotechnology). Then, the membrane was incubated with Goat anti-rabbit IgG (H + L)-HRP (1:5000; GenDEPOT, Baker, TX, USA) or m-IgGκ BP-HRP (1:5000; Santa Cruz Biotechnology) at room temperature for 2 h. Protein signals were detected using Pierce ECL Plus Western blotting substrate (Thermo Scientific™, Waltham, MA, USA), and the signals were captured and quantified using the ChemiDoc Imaging System and Image Lab software (version 6.0.0; Bio-Rad, Hercules, CA, USA).

### 2.11. Statistical Analysis

All data are presented as the mean ± standard deviation. Treatment group means were compared by one-way analysis of variance followed by post hoc Duncan’s multiple range tests. A *p* value of less than 0.05 was considered significant for all tests, and all calculations were performed using Statistix 8.1 (Statistix, Tallahassee, FL, USA). Pearson’s correlation analysis was conducted using Statistix 8.1.

## 3. Results

### 3.1. Antioxidant Activity of DJ526 Rice Callus Extract

The antioxidant activities of various concentrations of DJ526 rice callus extract were evaluated using the ABTS radical scavenging method (Table 3), comparing them with those of normal rice callus extract (DJ) and arbutin (positive control). Treatment with Dimethylsulfoxide (DMSO) at 0.1% (*v*/*v*) showed an ABTS radical scavenging level of 0.10% ± 0.09%, which did not significantly differ compared to the negative control. 

The ABTS radical scavenging activity increased with higher concentrations of DJ526 rice callus extract and arbutin. The antioxidant activity of DJ526 rice callus extract was also expressed in terms of Vitamin C Equivalent Antioxidant Capacity (VCEAC) using a standard curve of ABTS radical scavenging activity against ascorbic acid (vitamin C) concentration (Figure S1). 

Treatment with DJ526 rice callus extract at 100 mg/mL significantly enhanced antioxidant activity, similar to treatment with arbutin at 100 mg/mL. Moreover, at a concentration of 100 mg/mL, the antioxidant activity of DJ526 rice callus extract was equivalent to 0.237 ± 0.002 mg/g of vitamin C. The concentration of DJ526 rice callus extract required to reduce the initial ABTS radical concentration by 50% (IC50) was not within the calculable range (>100 mg/mL). For DJ526 rice callus extract and arbutin, the IC 50 values were 42.24 ± 0.67 mg/mL and 29.57 ± 0.55 mg/mL, respectively. A lower IC50 value indicates higher antioxidant activity. Therefore, DJ526 rice callus extract exhibits higher antioxidant activity than DJ rice callus extract.

**Figure 1:**
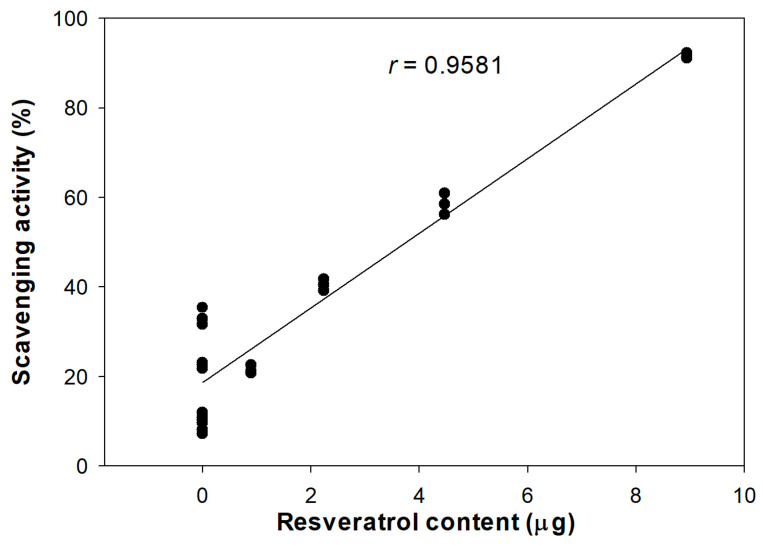
Pearson’s correlation analysis between the amount of resveratrol (piceid + resveratrol) and scavenging activity (degrees of freedom = 22, *p* = 0.05).

The antioxidant activity of DJ526 rice callus extract was strongly correlated with its resveratrol content (Figure 1). The Pearson’s correlation coefficient between antioxidant activity and resveratrol content was 0.9581 (*r*).

### 3.2. Melan-A Cell Viability Effect of DJ526 Rice Callus Extract

**Figure 2:**
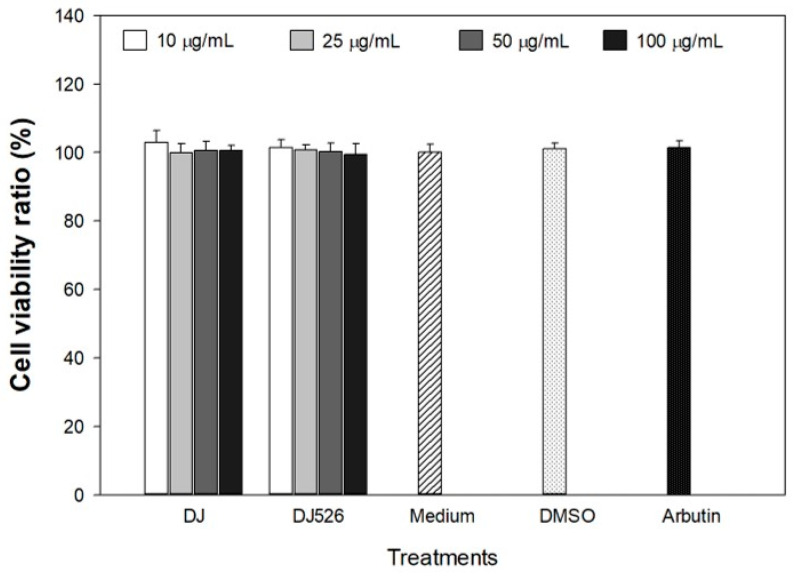
Effect of DJ526 rice callus extract on melan-a cell viability. The concentration of DMSO and arbutin was 0.1% (*v*/*v*) and 100 µg/mL, respectively. Data are shown as mean ± standard deviation (n = 3). Significant differences at *p* < 0.05 when compared with medium group.

The rice callus extracts were evaluated for cytotoxicity on melan-a cells before proceeding with further cell experiments. Extracts at concentrations of 10, 25, 50, and 100 µg/mL were applied to melan-a cells, and the cell viability for each treatment was compared with that of medium-treated cells. We observed no significant difference in melan-a cell viability (*p* < 0.05) (Figure 2), indicating that treatment with 0.1% (*v*/*v*) DMSO, 100 µg/mL of arbutin, and various concentrations (10, 25, 50, and 100 µg/mL) of DJ and DJ526 rice callus extracts has no cytotoxic effect on melan-a cells. Therefore, these concentrations of DJ and DJ526 rice callus extracts are suitable for use in melan-a cell experiments.

### 3.3. Melanin Content and Cellular Tyrosinase Activity Effect of DJ526 Rice Callus Extract 

**Figure 3:**
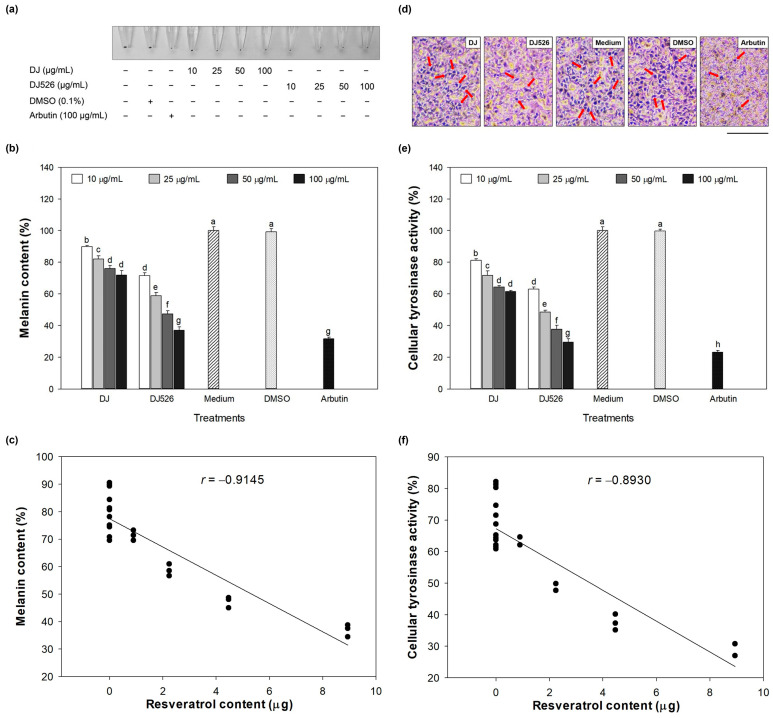
Effect of DJ526 rice callus extract on (**a**,**b**) melanin content, (**c**) Pearson’s correlation analysis between the amount of resveratrol (piceid + resveratrol) and melanin content (degrees of freedom = 22, *p* = 0.05), (**d**) representative L-DOPA staining at 100 µg/mL of treatments (scale bar = 50 µm), (**e**) cellular tyrosinase activity, and (**f**) Pearson’s correlation analysis between the amount of resveratrol (piceid + resveratrol) and cellular tyrosinase activity (degrees of freedom = 22, *p* = 0.05). The concentration of DMSO and arbutin was 0.1% (*v*/*v*) and 100 µg/mL, respectively. Data are presented as mean ± standard deviation (n = 3). Significant differences at *p* < 0.05. Lowercase letters (a–h) indicate significant differences at *p* < 0.05 among all treatments. In the statistical analysis, “a” represents the reference group, “b” represents significantly lower than the “a” group (*p* < 0.05), and “c” represents significantly lower than the “b” group (*p* < 0.05). Red arrows represent the melanin-containing cells.

After culturing melan-a cells with 10–100 µg/mL of treatments for 72 h, cells were collected, and melanin content in the treated melan-a cells was measured. There was no significant difference in melanin content between DMSO- and medium-treated cells (*p* < 0.05) (Figure 3a,b). However, the melanin content was significantly reduced in DJ- and DJ526-treated cells compared to medium-treated cells (*p* < 0.05). At the same concentration, the melanin content of DJ526-treated cells was significantly lower than that of DJ-treated cells (*p* < 0.05). Moreover, at 100 µg/mL, DJ526 showed the highest reduction in melanin content, achieving a level comparable to that of arbutin (positive control). Additionally, the reduction in melanin content by DJ526 rice callus extract significantly correlated with its resveratrol content (Figure 3c).

L-DOPA staining was used to indicate the melanin-containing cells. Figure 3d shows dark spots representing melanin-containing cells, which were primarily observed in the medium and DMSO groups. Treatment with DJ decreased the dark spots compared to the medium group. However, dark spots were dramatically reduced by DJ526 and arbutin compared to DJ. L-DOPA is catalyzed by tyrosinase during melanogenesis [50]. Therefore, cellular tyrosinase activity was evaluated to confirm the reduction in dark spots. Figure 3e shows that treatment with DJ and DJ526 rice callus extracts at concentrations of 10–100 µg/mL significantly decreased (*p* < 0.05) tyrosinase activity compared to the medium group. At 100 µg/mL, the reduction in cellular tyrosinase activity with DJ526 rice callus extract and arbutin was 52.04 ± 3.53% (decreasing from 61.51% ± 0.63% to 29.50% ± 2.17%) and 62.59 ± 2.12% (decreasing from 61.51% ± 0.63% to 23.01% ± 1.13%) compared to DJ. Pearson’s correlation analysis shows that the reduction in cellular tyrosinase activity was related to the content of resveratrol in DJ526 rice callus extract (*r* = −0.8930; Figure 3f). Therefore, the decrease in cellular tyrosinase activity in melan-a cells is attributed to the resveratrol content in DJ526 rice callus extract.

### 3.4. Effect of DJ526 Rice Callus Extract on Melanin Content and Morphological Appearance of Melan-A Cells 

**Figure 4:**
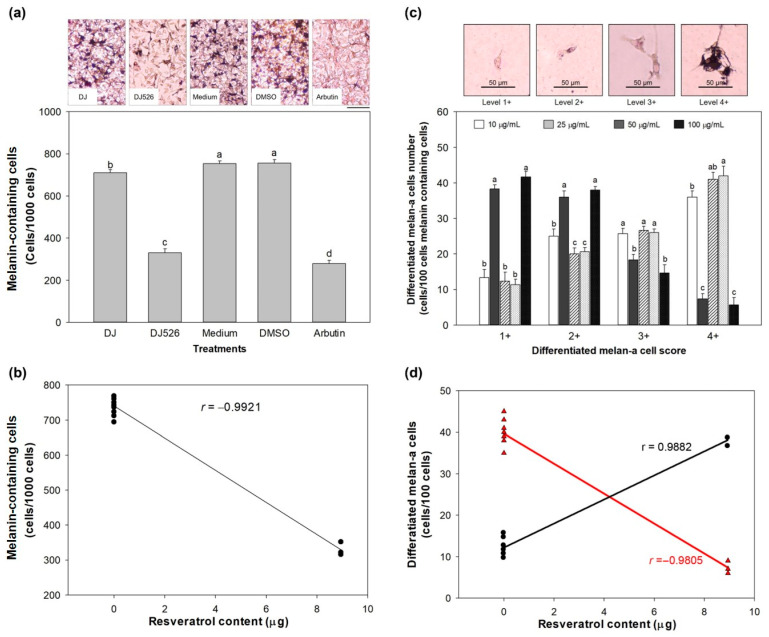
Effect of DJ526 rice callus extract on (**a**) melanin-containing cells (scale bar = 50 µm), (**b**) morphological appearance of melan-a cells, (**c**) Pearson’s correlation analysis between the amount of resveratrol (piceid + resveratrol) and melanin-containing cells (degrees of freedom = 10, *p* = 0.05), and (**d**) Pearson’s correlation analysis between the amount of resveratrol (piceid + resveratrol) and differentiated melan-a cells scored 4+ (Δ) and 1+ (•) (degrees of freedom = 10, *p* = 0.05). The concentration of arbutin, DJ, and DJ526 rice callus extracts was 100 µg/mL. The concentration of DMSO was 0.1% (*v*/*v*). Lowercase letters (a–d) indicate significant differences at *p* < 0.05 among treatments. In the statistical analysis, “a” represents the reference group, “b” represents significantly lower than the “a” group (*p* < 0.05), “c” represents significantly lower than the “b” group (*p* < 0.05), and “d” represents significantly lower than the “c” group (*p* < 0.05).

After culturing the cells with the treatments for 72 h, the cells were stained using the Fontana-Masson kit to visualize melanin pigment in melan-a cells as dark spots. The number of melanin-containing cells was recorded by randomly counting 1000 cells (Figure 4a). Treatment with DJ rice callus extract at 100 µg/mL significantly decreased the number of melanin-containing cells compared to the medium or DMSO group. A dramatic decrease in melanin-containing cells was observed in DJ526- and arbutin-treated cells. Figure 4b shows the correlation between melanin-containing cells and the resveratrol content in DJ526 rice callus extract (*r* = −0.9921). The reduction in melanin-containing cells in DJ526-treated cells was 56.23 ± 2.56% and 53.54 ± 2.72% compared to the medium- and DJ-treated cells, respectively. 

Melanin-containing cells were categorized into four groups depending on their morphology. In the medium-treated cells, most cells were larger than 51 µm with pigmentation (level 3+) and densely packed pigmentation (level 4+) throughout the cells (Figure 4c). Treatment with DJ526 rice callus extract at 100 µg/mL remarkably decreased the number of differentiated melan-a cells at levels 3+ and 4+ compared to the medium group. 

Furthermore, DJ526 rice callus extract significantly enhanced the population of differentiated melan-a cells at levels 1+ and 2+, similar to the arbutin group. The increase in the cell score 1+ population (*r* = 0.9882) and reduction in the cell score 4+ population (*r* = −0.9805) were related to the resveratrol content in DJ526 rice callus extract (Figure 4d).

### 3.5. Effect of DJ526 Rice Callus Extract on Melanogenesis-Associated Gene Expression

**Figure 5:**
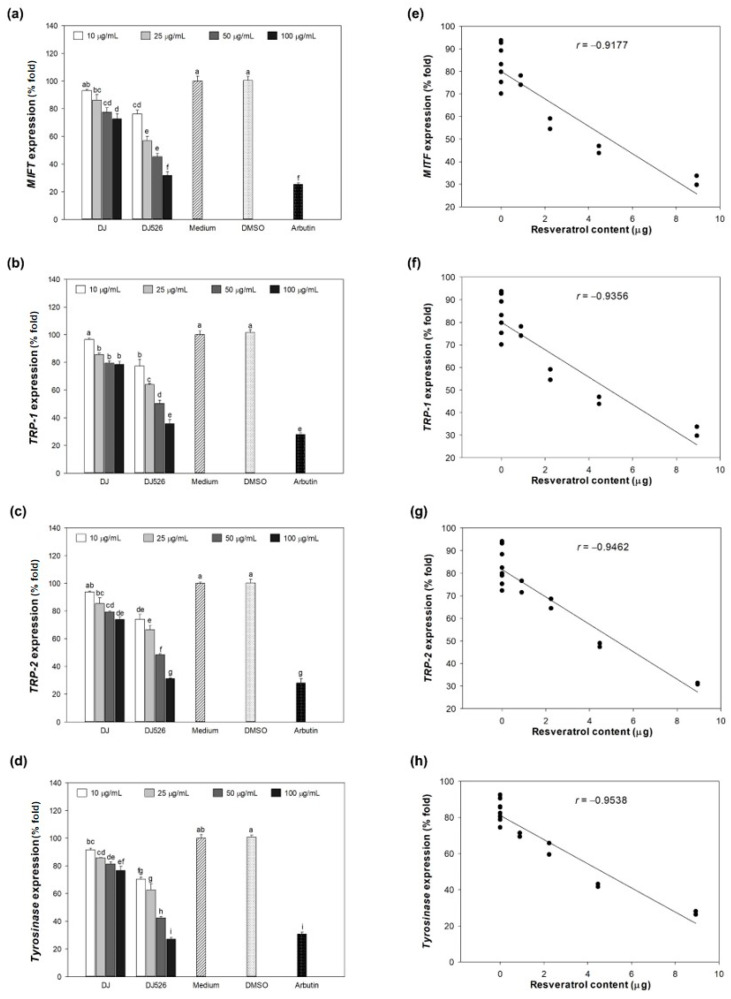
Effect of DJ526 rice callus extract on melanogenesis-associated gene expression. Expression levels of (**a**) *MITF*, (**b**) *TRP-1*, (**c**) *TRP-2*, and (**d**) *tyrosinase*. Pearson’s correlation analyses between the amount of resveratrol (piceid + resveratrol) and (**e**) *MITF*, (**f**) *TRP-1*, (**g**) *TRP-2*, and (**h**) *tyrosinase* expression levels. The concentration of DMSO and arbutin was 0.1% (*v*/*v*) and 100 µg/mL, respectively. Data are shown as mean ± standard deviation. Pearson’s correlation analyses were performed with degrees of freedom = 10 and *p* = 0.05. Significant differences at *p* < 0.05. Lowercase letters (a–i) indicate significant differences at *p* < 0.05 among all treatments. In the statistical analysis, “a” represents the reference group, “b” represents significantly lower than the “a” group (*p* < 0.05), “c” represents significantly lower than the “b” group (p < 0.05), “d” represents significantly lower than the “c” group (*p* < 0.05), “e” represents significantly lower than the “d” group (*p* < 0.05), “f” represents significantly lower than the “e” group (*p* < 0.05), “g” represents significantly lower than the “f” group (*p* < 0.05), “h” represents significantly lower than the “g” group (*p* < 0.05), and “i” represents significantly lower than the “h” group (*p* < 0.05).

The expression levels of melanogenesis-related mRNA in the control group (medium), including *MITF*, *TRP-1*, *TRP-2*, and *tyrosinase*, were assigned as 100% expression (Figure 5a–d). Treatment with 0.1% (*v*/*v*) DMSO did not show a significant difference compared to the control group. However, treatment with rice callus extracts remarkably decreased the expression levels of those genes compared to the control group. The expression levels of melanogenesis-related mRNAs decreased in a concentration-dependent manner with the extracts (DJ and DJ526 rice callus extract). Compared with DJ rice callus extract, DJ526-treated cells effectively downregulated the expression levels of those mRNAs. Pearson’s correlation analysis showed that the reduction in levels of melanogenesis-related genes was related to the amount of resveratrol content in DJ526 rice callus extract (Figure 5e–h). 

### 3.6. Effect of DJ526 Rice Callus Extract on Protein Expression

**Figure 6:**
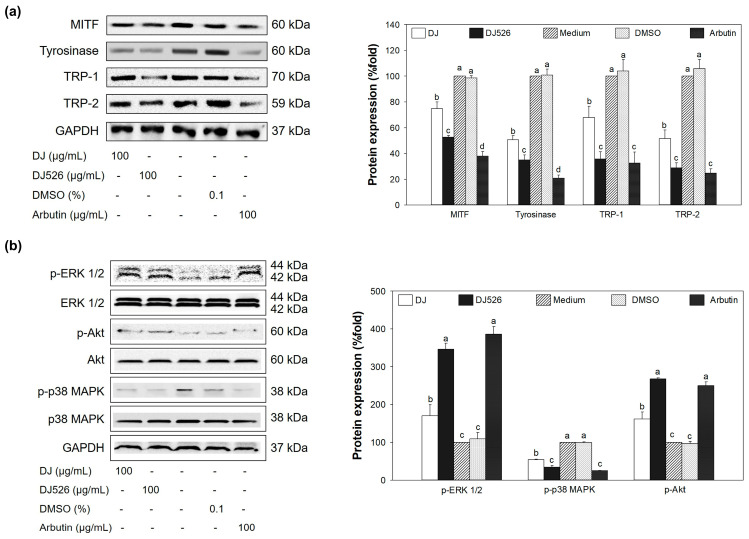
Effect of DJ526 rice callus extract on (**a**) melanogenesis-related protein expression and (**b**) inflammatory-related protein expression. Data are presented as mean ± standard deviation. Significant differences at *p* < 0.05. Lowercase letters (a–d) indicate significant differences at *p* < 0.05 among the treatments. In the statistical analysis, “a” represents the reference group, “b” represents significantly lower than the “a” group (*p* < 0.05), “c” represents significantly lower than the “b” group (*p* < 0.05), and “d” represents significantly lower than the “c” group (*p* < 0.05).

The highest expression level of MITF protein was observed in medium- and DMSO-treated cells (Figure 6a), resulting in high levels of TRP-1, TRP-2, and tyrosinase protein expression. Treatment with 100 µg/mL of DJ526 rice callus extract markedly downregulated MITF protein expression compared to medium and DJ rice callus extract (100 µg/mL). The downregulation of MITF by DJ526 rice callus extract led to decreased expression of TRP-1, TRP-2, and tyrosinase proteins. Additionally, MITF regulation is also associated with the regulation of MAPK and PI3K/Akt signaling pathways [51]. Figure 6b shows that DJ526 rice callus extract effectively upregulated the expression levels of p-ERK 1/2 and p-Akt compared to medium, DMSO, and DJ rice callus extract. However, the expression level of p-p38 MAPK protein was reduced by treatment with DJ526 rice callus extract compared to medium, DMSO, and DJ rice callus extract. These results indicate that melanogenesis in melan-a cells was suppressed by treatment with DJ526 rice callus extract through inhibition of MITF protein expression, resulting in reduced levels of TRP-1, TRP-2, tyrosinase, and p-p38 MAPK proteins, and increased levels of p-ERK 1/2 and p-Akt. 

## 4. Discussion

This study demonstrated that DJ526 rice callus extract, containing high resveratrol content, enhances antioxidant activity by promoting ABTS radical scavenging ability compared to DJ rice callus extract. This inhibitory effect significantly correlated with the amount of resveratrol content in DJ526 rice callus extract (Figure 1). This result is supported by the report of Li et al. [52], which suggested that resveratrol extracted from mulberry root (*Morus alba* L.) shows high antioxidant potential by improving ABTS radical scavenging ability. The incorporation of alcoholic beverages (red wine, white wine, and tsipouro) and resveratrol significantly increased ABTS radical scavenging ability [53]. 

Melanogenesis is a key pathway for melanin synthesis [54]. The important transcription factor in this pathway is MITF, which is associated with the regulation of TRP-1, TRP-2, and tyrosinase [55,56]. Tyrosinase is the key enzyme that hydroxylates L-tyrosine to L-DOPA and L-DOPA, which is then oxidized to DOPA-quinone by tyrosinase as well [57]. The production of melanin requires TRP-1 and TRP-2 to synthesize eumelanin, which is the most common form of melanin (brown to black) [58,59]. Saha et al. [60] reported that the activation of p38 MAPK by UV results in the promotion of MITF [61]. Conversely, the activation of ERK 1/2 and Akt enhances the degradation of MITF, causing the downregulation in the expression and activity of tyrosinase [62,63]. Although melanin synthesis is important for protecting the skin from external stimuli including UV [64], excessive production can lead to hyperpigmentation [13]. 

This study showed that treatment with resveratrol-enriched rice callus extract (DJ526) significantly suppressed the expression of tyrosinase, TRP-1, and TRP-2 by inhibiting the production of MITF at both mRNA and protein levels (Figure 5 and Figure 6a). Additionally, DJ526 markedly reduced the activation of the MITF promoter (p-p38 MAPK) and enhanced the activation of MITF degradation inducers (p-ERK 1/2 and p-Akt) (Figure 6b). These results are supported by the report of Mansky et al. [65], which demonstrated the correlation between the activation of MITF and p-p38 MAPK. Similarly, Tagashira et al. [66] showed that upregulation of p-p38 MAPK expression by UVB exposure leads to increased MITF and melanocyte-specific protein expression. Bhat et al. [67] reported the reduction in MITF, tyrosinase, TRP-1, and TRP-2 expression by blocking the modulation of p-p38 MAPK. Kim and Hyun [68] suggested that reduction in p-Akt and p-ERK 1/2 promote the expression of tyrosinase, melanin content, and pigmentation induction. This is similar to the study of Huang et al. [69], which confirmed the inhibition of melanogenesis through the activation of ERK 1/2 and Akt-mediated MITF degradation. Additionally, several studies have demonstrated the potential of resveratrol in suppressing melanogenesis. Treatment of resveratrol extracted from *Veratrum album* var. *grandiflorum* significantly decreased the activity of tyrosinase in murine melanoma B-16 cells [35]. Oxyresveratrol extracted from *Morus australis* stems exhibits strong tyrosinase inhibitory activities [37]. Lee et al. [70] showed that resveratrol-enriched rice extract significantly reduced UVB-induced hyperpigmentation on the skin of guinea pigs. 

The inhibitory effect of resveratrol-enriched rice callus extract on melanogenesis also affected the number of melanin-containing cells, and the size and morphological appearance of melan-a cells (Figure 3 and Figure 4). Resveratrol inhibits the growth and induces apoptosis of human melanoma A375 [71,72], MV3 cells [71], and SK-MEL-31 [72]. Moreover, resveratrol decreased the production of melanin (melanin content) in B16 melanoma cells [73]. Yu et al. [74] reported that resveratrol significantly inhibits the colony formation and migration of mouse melanoma B16 cells.

## 5. Conclusions

We demonstrate that resveratrol-enriched rice callus extract (DJ526) potentially exhibits antioxidative and anti-melanogenic effects on melan-a cells. DJ526 suppressed the expression of MITF by promoting the activation of MITF degradation inducers (p-ERK 1/2 and p-Akt) and by suppressing the activation of the MITF promoter (p-p38 MAPK), resulting in the downregulation of tyrosinase, TRP-1, and TRP-2 mRNA and protein levels. The reduction in MITF expression directly affected melanin production in the melanogenesis process. DJ526 significantly decreased the size and altered the melanin distribution of melan-a cells. These findings demonstrate that the resveratrol contained in DJ526 rice seeds is further enriched by callus induction, and resveratrol-enriched rice callus may be considered a potentially useful natural agent for controlling hyperpigmentation.

## Figures and Tables

**Table 1:** Quantity and quality of total RNA.

Treatment	A260:A280	A260:A230	RNA Concentration (ng/µL)		
			Mean	SD	CV
Medium	1.832	1.962	306.45	29.75	9.71
DMSO (0.1%)	1.947	1.831	465.17	34.16	7.34
Arbutin (100 µg/mL)	1.981	1.998	768.59	21.54	2.80
DJ (10 µg/mL)	1.894	1.890	268.20	19.72	7.35
DJ (25 µg/mL)	2.001	1.994	474.80	44.15	9.30
DJ (50 µg/mL)	1.989	1.891	647.25	53.72	8.30
DJ (100 µg/mL)	1.865	1.881	574.19	21.74	3.79
DJ526 (10 µg/mL)	1.975	1.938	640.72	10.19	1.59
DJ526 (25 µg/mL)	1.968	1.966	650.67	55.29	8.50
DJ526 (50 µg/mL)	1.942	1.947	143.60	2.70	1.88
DJ526 (100 µg/mL)	1.835	1.959	791.73	12.77	1.61

Note: A260:A280 represents the ratio of absorbance at 260 nm to that at 280 nm. A260:A230 represents the ratio of absorbance at 260 nm to that at 230 nm. SD represents the standard deviation (n = 3). CV represents the coefficient of variation.

**Table 2:** qPCR conditions used for quantifying gene expression.

Process	Temperature (°C)	Time (min:s)	Number of Cycles
Pre-denaturation	95	10:00	1
PCR cycle - Denaturation - Annealing - Extension	95 60 72	0:20 0:20 0:30	40
Final extension	72	5:00	1
Melt curve	65–95	Every 0:05	1

**Table 3:** Scavenging activity and vitamin C equivalent antioxidant capacity of DJ526 rice callus extract.

Treatment	Concentration	Scavenging Activity (%)		VCEAC (mg/g)		IC50 (mg/mL)
		Mean	SD	Mean	SD	
Control (DW)	−	0.00	0.61	0.000	0.002	−
DMSO	0.1% (*v*/*v*)	0.10	0.09	0.000	0.000	−
DJ	10 mg/mL	8.34 ^c^	1.21	0.004 ^c^	0.003	Noncalculable
	25 mg/mL	10.99 ^c^	0.83	0.012 ^c^	0.002	
	50 mg/mL	22.48 ^c^	0.68	0.044 ^c^	0.002	
	100 mg/mL	33.32 ^c^	1.91	0.074 ^c^	0.005	
DJ526	10 mg/mL	21.48 ^b^	0.94	0.041 ^b^	0.003	42.24 ± 0.67
	25 mg/mL	40.46 ^b^	1.27	0.094 ^b^	0.004	
	50 mg/mL	58.54 ^b^	2.32	0.145 ^b^	0.006	
	100 mg/mL	91.66 ^a^	0.62	0.237 ^a^	0.002	
Arbutin	10 mg/mL	25.32 ^a^	0.62	0.052 ^a^	0.002	29.57 ± 0.55
	25 mg/mL	50.80 ^a^	0.74	0.123 ^a^	0.002	
	50 mg/mL	77.77 ^a^	1.26	0.198 ^a^	0.004	
	100 mg/mL	93.71 ^a^	0.40	0.243 ^a^	0.001	

Note: SD represents the standard deviation (n = 3). VCEAC represents the vitamin C (ascorbic acid) equivalent antioxidant capacity. Lowercase letters (a–c) indicate significant differences at *p* < 0.05 among treatments at the same concentration. In the statistical analysis, “a” represents the reference group, “b” represents significantly lower than the “a” group (*p* < 0.05), and “c” represents significantly lower than the “b” group (*p* < 0.05).

## Data Availability

All data generated for this project are included in the manuscript. The authors will provide additional details upon reasonable request.

## Supplementary Materials

The following supporting information can be downloaded at: https://www.mdpi.com/article/10.3390/antiox13060625/s1, Figure S1: Standard curve of ABTS radical scavenging activity plotted against varied concentrations of vitamin C; Figure S2: Standard curve for bovine serum albumin; Table S1: Differentiated melan-a cell scoring.
