# Supplementary material for: Resveratrol-Enriched Rice Callus Extract Inhibits Oxidative and Cellular Melanogenic Activities in Melan-A Cells
Source: Antioxidants (Basel). 2024 May 21;13(6):625. doi: 10.3390/antiox13060625 (PMC11201182; doi:10.3390/antiox13060625)
Supplement: Supplementary file 1 [file antioxidants-13-00625-s001.zip › antioxidants-3026601-supplementary.pdf]

Supplementary materials

Figure S1. Standard curve of ABTS radical scavenging activity plotted against varied concentrations of vitamin C.

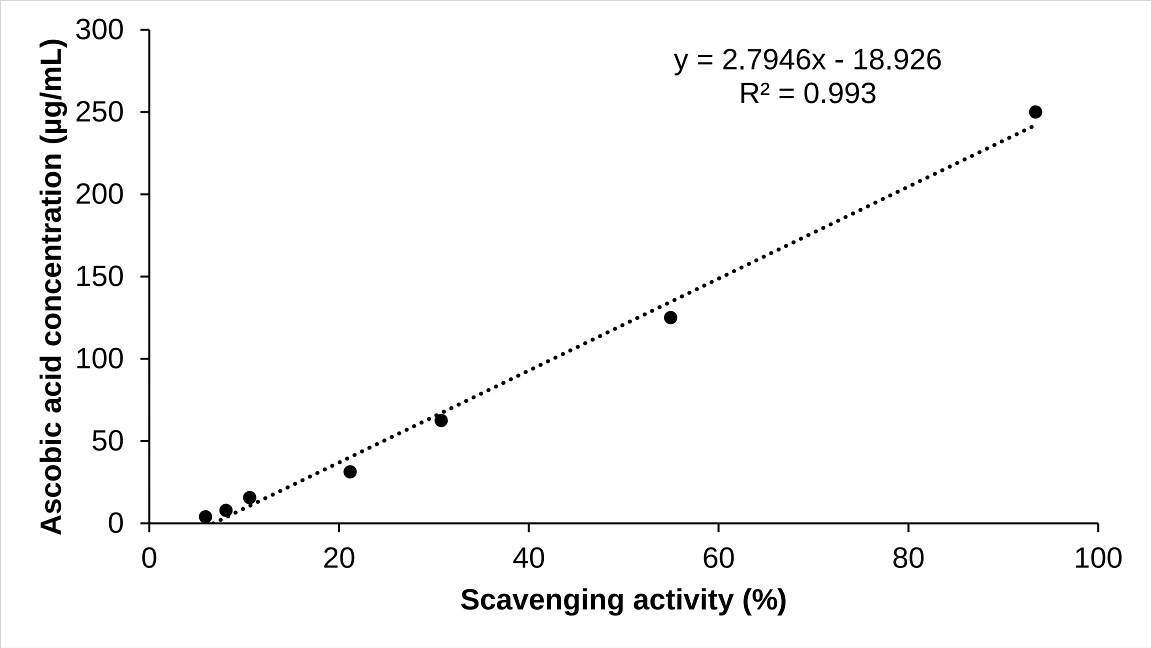

Figure S2. Standard curve for bovine serum albumin

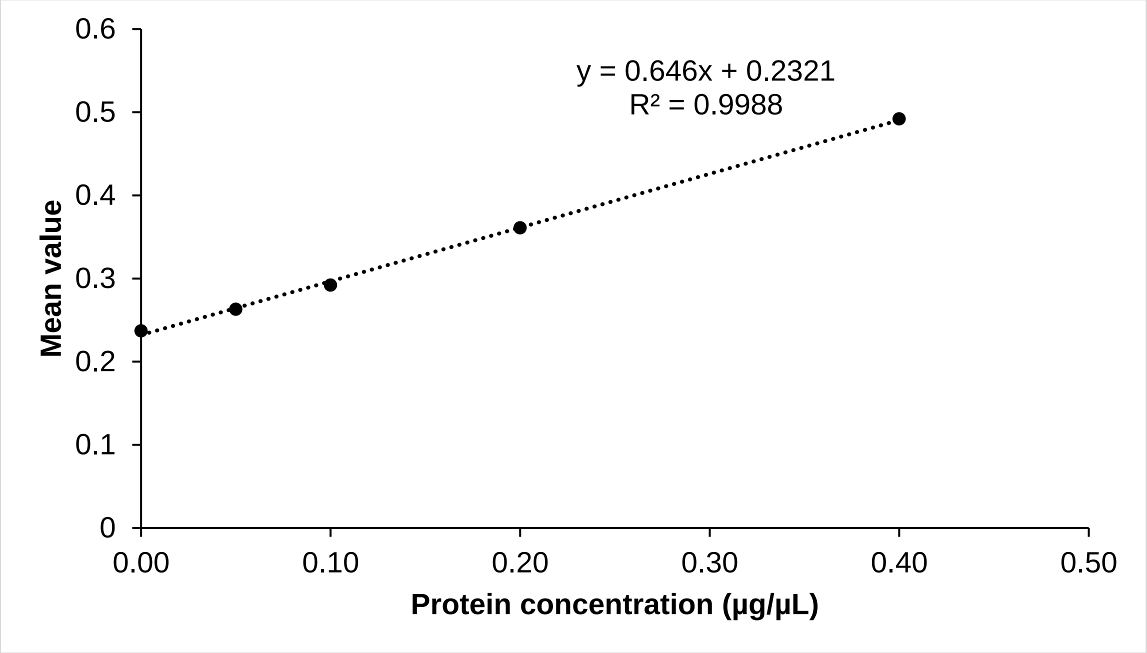

Table S1. Differentiated melan-a cell scoring

Benchmark	Score
Cell size lower than 50 µm with pigmentation lower than 50% of cytoplasmic area with a low distribution.	1+
Cell size lower than 50 µm with pigmentation equal or higher than 50% of cytoplasmic area with a higher distribution.	2+
Cell size larger than 51 µm with pigmentation throughout the cytoplasmic area.	3+
Cell size larger than 51 µm with densely packed pigmentation throughout the cytoplasmic area.	4+
